# CTRP-3 em Pacientes com Doença Arterial Coronariana Estável e Fibrilação Atrial Paroxística: Um Novo Biomarcador Potencial nas Doenças Cardiovasculares

**DOI:** 10.36660/abc.20210940

**Published:** 2022-01-01

**Affiliations:** 1 Universidade do Estado do Rio de Janeiro Rio de Janeiro RJ Brasil Universidade do Estado do Rio de Janeiro – Cardiologia, Rio de Janeiro, RJ – Brasil; 2 Américas Serviços Médicos Hospital Pró-Cardíaco Rio de Janeiro RJ Brasil Hospital Pró-Cardíaco – Américas Serviços Médicos, Rio de Janeiro, RJ – Brasil

**Keywords:** C1q (Fatores Imunológicos), Fatores de Necrose Tumoral, Adiponectina, Anti-Inflamatórios, Doença da Artéria Coronariana, Fatores de Risco, Fibrilação Atrial

A família de proteínas relacionadas ao fator de necrose tumoral C1q (CTRP), composta por 15 membros (CTRP1-CTRP15), é um parálogo recentemente descoberto e altamente conservado da adiponectina. Embora a família CTRP e a adiponectina apresentem similaridades estruturais, elas exercem efeito pleiotrópico sobre o metabolismo celular e apresentam diferentes padrões regulatórios.^1-3^ A CTRP3, membro dessa família, é uma potente adipocina anti-inflamatória que inibe vias pró-inflamatórias em monócitos e microcélulas, exercendo um efeito anti-inflamatório, anti-apoptótico, e cardioprotetor durante o desenvolvimento da doença arterial coronariana (DAC).^3-5^ Ainda, essa adiponectina tem propriedades cardioprotetoras, e uma associação inversa com parâmetros de resistência insulínica; seus níveis circulantes diminuem na obesidade e na hipertensão.^6^

Um dos primeiros estudos a avaliar níveis circulantes de CTRP-3 e progranulina em pacientes com DAC foi conduzido na Coreia, com 362 adultos com síndrome coronária aguda (SCA) e angina estável, e indivíduos controle com vários fatores de risco cardiometabólicos. As concentrações de CTRP-3 encontravam-se significativamente reduzidos em pacientes com SCA ou angina estável em comparação aos controles. Análise de correlação, ajustada por idade e sexo, revelou que os níveis de CTRP-3 apresentaram uma relação negativa significativa com níveis de glicose e proteína C reativa, e uma relação positiva com níveis de HDL e adiponectina. Na análise de regressão logística multivariada, o *odds ratio* para DAC foi 5,14 no segundo tercil, e 9,04 no primeiro tercil dos níveis de CTRP-3 em comparação ao terceiro tercil, após ajuste para as variáveis de risco cardiometabólico. Tais resultados sugerem que CTRP-3 possa ser útil na avaliação de risco para DAC.^7^

Fadaei et al.,^8^ determinaram os níveis séricos de CTRP-3, CTRP-13, adiponectina e citocinas inflamatórias, e sua expressão gênica em células mononucleares no sangue periférico em 172 indivíduos categorizados como grupo I (sem diabetes mellitus tipo 2 [DM2] e DAC, grupo II [com DAC e sem DM2], grupo III [com DM2 e sem DAC] e grupo IV [com DM2 e DAC]). Os níveis séricos e a expressão gênica de CTRP-3, CTRP-13, e adiponectina no grupo I foram maiores em comparação aos outros grupos. Os níveis de CTRP-3 associaram-se de maneira independente com IMC, tabagismo e expressão gênica de CTRP-3. Níveis reduzidos de CTRP-3 e CTRP-13 também apresentaram associação com DAC. Níveis diminuídos de CTRP-3, particularmente CTRP-13, parecem estar associados com risco aumentado de DM2 e DAC. A CTRP-3 apresentou uma associação negativa e independente significativa com DAC na população total do estudo.^8^

No estudo de Wang et al.,^9^ 145 pacientes que se submeteram à angiografia coronária foram divididos em dois grupos: sem DAC e com DAC. O grupo com DAC foi ainda dividido em três grupos: pacientes com doença de um, dois ou três vasos. Os níveis de CTRP-3 foram significativamente mais altos em pacientes com DAC que em pacientes sem DAC. Diferenças significativas de CTRP-3 também foram encontradas entre os pacientes com doença em dois vasos e em pacientes com doença em três vasos. Análise de regressão logística múltipla revelou que os níveis de CTRP-3, juntamente com colesterol HDL e glicose, correlacionaram-se com DAC.^9^

Ahmed et al.^10^ mediram marcadores bioquímicos e níveis séricos de CTRPs e MCP-1 em 86 mulheres pós-menopausa. As participantes foram divididas em quatro grupos: 13 aparentemente sadios como controle (grupo 1), 29 pacientes com DAC (grupo II), 29 pacientes com DM2 há mais de cinco anos, e 15 pacientes com DAC secundária a DM2 (grupo IV). Níveis séricos de CTRP-3 eram significativamente mais altos nos grupos III e IV, e significativamente mais baixos no grupo II em comparação ao grupo I. As duas CTRPs apresentaram correlação significativa negativa entre si.^10^

No cenário da fibrilação atrial (FA), não há estudo relatando se a CTRP-3 pode ter um papel na FA e remodelação atrial concomitante. Chen et al.,^11^ estudaram 75 pacientes com FA que se submeteram à ablação por cateter e 47 pacientes com ritmo sinusal e encontraram que as concentrações plasmáticas de CTRP-3 foram significativamente mais baixas em pacientes com FA em comparação ao grupo controle. Em estudos com subgrupos de pacientes, aqueles com FA persistente apresentaram concentrações mais baixas de CTRP-3 que pacientes com FA paroxística. As concentrações plasmáticas de CTRP-3 no grupo com recorrência após ablação por radiofrequência da FA foram mais baixas que no grupo sem recorrência. Análise de regressão multivariada revelou uma correlação independente entre CTRP-3 e FA.^11^

Na presente edição dos Arquivos Brasileiros de Cardiologia, no artigo de Yildirim et al.,^12^ 252 pacientes com DAC e 50 controles sadios foram divididos em grupos de pacientes com e sem DAC, e grupos de pacientes com DAC e FA paroxística e com DAC sem FA paroxística. Os níveis séricos de CTRP-3 foram significativamente mais baixos em pacientes com DAC que no grupo controle. A FA foi detectada em 15,08% do grupo com DAC. A frequência de hipertensão, indivíduos do sexo feminino, proteína C-reativa ultrassensível, nitrogênio ureico no sangue, níveis de creatinina e diâmetro diastólico do átrio esquerdo foram maiores, e os níveis de CTRP-3 foram mais baixos em pacientes com FA. Neste estudo, cada redução de 1ng/mL nos níveis de CTRP-3 aumentou o risco de FA em 10,7%. Na análise ROC dos valores de CTRP-3 para detectar pacientes com FA, a área sob a curva ROC para CTRP-3 foi 0,971, considerada estatisticamente significativa. Um ponto de corte de 300 ng/mL apresentou uma sensibilidade de 87,9% e especificidade de 86,8% para a presença de FA. Os autores concluíram que níveis séricos de CTRP-3 caíram significativamente em pacientes com DAC estável, e níveis reduzidos de CTRP-3 relacionaram-se com a presença de FA nesses pacientes.^12^ Esta é a primeira publicação que tenta associar os níveis de CTRP-3 com DAC e FA, necessitando de mais estudos para determinar a real importância dessa informação.^12^

CTRP-3 é um fator independente de FA e, de certa forma, a proteína está relacionada ao prognóstico dos pacientes com FA após ablação com radiofrequência.^11^

A família CTRP exerce um importante papel em todos os estágios da DAC pela regulação imune, de inflamação, do metabolismo da glicose e de lipídios, e função endotelial vascular. A CTRP-1 representa marcadores pró-inflamatórios e pró-ateroscleróticos ao contribuir para a secreção de citocinas inflamatórias e moléculas de adesão e promover a formação de células espumosas a partir de macrófagos. A CTRP-5 promove o crescimento, a migração e a inflamação de células musculares lisas vasculares. Por outro lado, CTRP-3, CTRP-9, CTRP-12, e CTRP-13 ativam mecanismos anti-inflamatórios e anti-ateroscleróticos da DAC, por inibição da inflamação endotelial e redução da formação de placas.^13,14^

Resultados positivos dessa investigação e maior conhecimento dos mecanismos moleculares possibilitarão a inclusão desses biomarcadores às diretrizes da DAC e FA.^3^
