# Supplementary material for: CTRP-3 em Pacientes com Doença Arterial Coronariana Estável e Fibrilação Atrial Paroxística: Um Novo Biomarcador Potencial nas Doenças Cardiovasculares
Source: Arq Bras Cardiol. 2022 Jan 1;118(1):59–60. [Article in Portuguese] doi: 10.36660/abc.20210940 (PMC8959052; doi:10.36660/abc.20210940)
Supplement: Translation

# Translation: CTRP-3 Levels in Patients with Stable Coronary Artery Disease and Paroxysmal Atrial Fibrillation: A New Potential Biomarker in Cardiovascular Diseases

**Affiliations:** 1 Rio de Janeiro RJ Brazil Universidade do Estado do Rio de Janeiro - Cardiologia, Rio de Janeiro, RJ - Brazil; 2 Rio de Janeiro RJ Brazil Hospital Pró-Cardíaco - Américas Serviços Médicos, Rio de Janeiro, RJ - Brazil

**Keywords:** C1q (Immunologic Factors), Tumor Necrosis Factors, Adiponectin, Anti-Inflammatory Agents, Coronary Artery Disease, Risk Factors, Atrial Fibrillation

The C1q tumor necrosis factor-related protein (CTRP) family, comprised of 15 members (CTRP1-CTRP15), is a newly discovered and highly conserved paralogue of adiponectin. Despite structural similarities between CTRP family and adiponectin, they exert pleiotropic effects on cell metabolism and have different regulation patterns.^1–3^ CTRP3 (also known as CORS-26, cartducin and cartonectin) is a member of this family. CTRP3 is a potent anti-inflammatory adipokine that inhibits proinflammatory pathways in monocytes and microcells, exerting anti-inflammatory, anti-apoptotic and cardioprotective effect during development of coronary artery disease (CAD).^3–5^ Also, this adipokine has cardioprotective properties and is inversely associated with insulin resistance parameters; its circulating levels drop in obesity and hypertension.^6^

One of the first studies to evaluate circulating CTRP-3 and progranulin levels in patients with CAD was conducted on 362 Korean adults with acute coronary syndrome (ACS), stable angina pectoris and control subjects with various cardiometabolic risk factors. CTRP-3 concentrations were significantly decreased in patients with ACS or stable angina compared to control subjects. Correlation analysis adjusted for age and gender showed that CTRP-3 levels had a significant negative relationship with glucose and high sensitive C-reactive protein levels, and a positive relationship with HDL-cholesterol and adiponectin levels. In a multivariate logistic regression analysis, the odds ratio for CAD was 5.14 in the second tertile, and 9.04 in the first tertile of CTRP-3 levels compared to the third tertile, after adjusting for other cardiometabolic risk variables. These results suggest that CTRP-3 might be useful for assessing the risk of CAD.^7^

Fadaei et al.^8^ determined serum levels of CTRP-3, CTRP-13, adiponectin and inflammatory cytokines and their gene expression in peripheral blood mononuclear cells in 172 subjects categorized as group I [without type 2 diabetes melitus (T2DM) and CAD], group II (with CAD but no T2DM), group III (with T2DM but no CAD) and group IV (with T2DM and CAD). Serum levels and gene expression of CTRP-3, CTRP-13 and adiponectin in the group I were higher compared to other groups. CTRP-3 serum levels had an independent association with BMI, smoking and CTRP-3 gene expression. Decreased serum levels of CTRP-3 and CTRP-13 were also associated with CAD. It appears that the decreased levels of CTRP-3, and particularly CTRP-13, were associated with increased risk of T2DM and CAD. CTRP-3 showed a significant independent negative association with the presence of CAD in the whole study population.^8^

In the study by Wang et al.,^9^ 145 patients who underwent coronary angiography were divided into two groups: non-CAD and CAD group. The CAD group was further divided into single-vessel, double-vessel, and triple-vessel disease groups. Serum levels of CTRP-3 were significantly higher in CAD patients than in non-CAD patients. Significant differences of CTRP 3 levels were also found between single-vessel group and triple-vessel group. Multiple logistic regression analysis revealed that CTRP 3 levels, together with HDL cholesterol and glucose, correlated with CAD.^9^

Ahmed et el.^10^ measured biochemical markers and serum levels of CTRPs and MCP-1 in 86 postmenopausal females. Subjects were divided into four groups: 13 apparent healthy subjects as control (group I), 29 patients with CAD (group II), 29 patients with T2DM for five years or longer (group III) and 15 patients with CAD secondary to T2DM (group IV). Serum CTRP-3 levels were found to be significantly higher in groups III and IV, and significantly lower in group II as compared with group I. Both CTRPs were significantly negatively correlated with each other.^10^

In the scenario of atrial fibrillation (AF), no study has reported whether CTRP-3 may play a role in AF and concomitant atrial remodeling. Chen et al.^11^ studied 75 AF patients who underwent catheter ablation and 47 sinus rhythm patients and found that plasma CTRP-3 concentrations were significantly lower in AF patients compared with control group. In subgroup studies, patients with persistent AF had lower plasma CTRP-3 concentrations than those with paroxysmal AF. The concentrations of plasma CTRP-3 in the recurrence group after radiofrequency catheter ablation of AF were lower than those in the nonrecurrence group. Multivariate regression analysis revealed the independent correlation between plasma CTRP-3 level and AF.^11^

In the present issue, Yildirim et al.^12^ studied 252 patients with CAD and 50 healthy controls, who were divided into groups with and without CAD and CAD patients with and without paroxysmal AF. Serum CTRP-3 levels were significantly lower in patients with CAD than in the control group. AF was detected in 15.08% of patients in the CAD group. The frequency of hypertension and female sex, high-sensitivity C-reactive protein, blood urea nitrogen, creatinine levels and left atrial diastolic diameter were higher, and CTRP-3 levels were lower in patients with AF. In this study, every 1 ng/mL reduction in CTRP-3 levels increased the risk of AF by 10.7%. In the ROC analysis of CTRP-3 values to detect patients with AF, the area under the ROC curve for CTRP-3 was 0.971 and considered statistically significant. A CTRP-3 cutoff point of 300 ng/mL had an 87.9% sensitivity and 86.8% specificity for the presence of AF. It was concluded that serum CTRP-3 levels dropped significantly in patients with stable CAD, and reduced levels of CTRP-3 were related to the presence of paroxysmal AF in these patients.^12^ This is the first publication that attempts to associate CTRP-3 levels with CAD and AF, requiring further studies to determine the real importance of this information.^12^

CTRP-3 is an independent factor of AF, and to some extent, it is related to the prognosis of patients with AF after radiofrequency ablation.^11^

The CTRP family plays an important role in all stages of CAD by regulating immuno-inflammation, glucose and lipid metabolism, and vascular endothelial function. CTRP-1 represents as pro-inflammatory and pro-atherosclerotic markers by contributing toward the secretion of inflammatory cytokines and adhesion molecules and promoting the formation of foam cells from macrophages. CTRP-5 promotes vascular smooth muscle cell growth, migration, and inflammation. In contrast, CTRP-3, CTRP-9, CTRP-12, and CTRP-13 activate anti-inflammatory and anti-atherosclerotic mechanisms of CAD, by inhibiting endothelial inflammation and reducing plaque formation.^13,14^

Positive results from such research and further understanding of their molecular mechanisms will promote adding these biomarkers to CAD and AF guidelines.^3^
